# Towards renewable hydrogen-based electrolysis: Alkaline vs Proton Exchange Membrane^☆^

**DOI:** 10.1016/j.heliyon.2023.e17999

**Published:** 2023-07-11

**Authors:** Bernhard N.D. van Haersma Buma, Marco Peretto, Ziad M. Matar, Geerten van de Kaa

**Affiliations:** Technology, Policy & Management, TU Delft, Jaffalaan 5, Delft 2628BX, the Netherlands

**Keywords:** Proton Exchange Membrane, Alkaline electrolysis, Technology dominance, Best worst method, Green hydrogen, Innovation

## Abstract

This paper focuses on the battle for a dominant design for renewable hydrogen electrolysis in which the designs, alkaline and proton exchange membrane, compete for dominance. First, a literature review is performed to determine the most relevant factors that influence technology dominance. Following that, a Best Worst Method analysis is conducted by interviewing multiple industry experts. The most important factors appear to be: Price, Safety, Energy consumption, Flexibility, Lifetime, Stack size and Materials used. The opinion of experts on Proton Exchange Membrane and alkaline electrolyser technologies is slightly skewed in favour of alkaline technologies. However, the margin is too small to identify a winner in this technology battle. The following paper contributes to the ongoing research on modelling the process of technology selection in the energy sector.

## Highlights

• Differentiating factors for technology dominance are identified.
• Using the Best Worst Method, factors are ranked by importance.
• Expert opinions are slightly skewed in favour of alkaline technologies.

## Nomenclature

**Acronyms & abbreviations**
**AEM**: Anion Exchange Membrane
**BWM**: Best Worst Method
**EU**: European Union
**MPa**: Megapascal
**MW**: Megawatt
**PEM**: Proton Exchange Membrane
**RED II**: Renewable Energy Directive II
**RES**: Renewable Energy Sources

## 1 Introduction

Hydrogen is a versatile molecule that can be used as feed stock in the industry or as an energy carrier. When burned, it only emits water, making it a great asset for the decarbonisation of industrial sectors where reduction of carbon emissions is both urgent and difficult [1]. However, hydrogen is not always produced in a renewable way. Currently around 95% of hydrogen produced worldwide is made using natural gas or coal, resulting in large CO_2_ emissions [2]. Hydrogen produced from fossil sources such as coal and natural gas is often called grey hydrogen. The green alternative for grey hydrogen is renewable hydrogen, made using renewable electricity and electrolysers. In electrolysis water is split into hydrogen and oxygen by directing an electric current through the molecules. Between the 1920s and the 1980s this technology was widespread, but eventually could not compete with cheaper hydrogen from fossil sources [3].

In recent years, renewable hydrogen has been making a comeback, and could be one of the most crucial components of the energy transition [4]. The Hydrogen Roadmap Europe states that renewable hydrogen could account for 24% of the total EU energy demand [5]. It is predicted that in 2030 there will be an available electrolyser capacity of 40 GW across Europe. In the electrolyser market, a battle for dominance is being waged between two main rival technologies: Alkaline and Proton Exchange Membrane (PEM). This research will focus on this battle for a dominant design. Alkaline technology is the older technology which has not been developed substantially over the last decades. Nonetheless, it is commonly utilised in the chlorine industry. Most chlorine is produced by electrolysis operations that consume brine instead of water. In fact, electrolysis production underpins more than 55% of the European chemical industry [6]. Some established chlorine industry players are now making the move from brine electrolysis to alkaline based water electrolysis. PEM electrolysis is a newer technology that has been specifically developed to produce hydrogen and to overcome issues with alkaline electrolysers [7].

Currently, there are about as many adopters of PEM technology as there are of alkaline technology and the question is whether one technology will become dominant and if so which one. Technology management and standardisation scholars have studied multiple similar battles [5], [8], [9], [10], and arrived at factors for technology dominance [11], [12], [13], [14] which may by applied for this specific case to better understand why one dominant standard has not been reached. Additionally, by consulting industry and academia, it is possible to understand which factors affect dominance in the field of electrolysers. This leads to the following research question: Which factors affect technology dominance for renewable hydrogen-based electrolysis according to experts?

We aim to contribute to the literature on standardization and dominant designs by investigating the extent to which factors for technology dominance can be determined and its process modelled. This has been done in the past by investigating various cases in the energy sector [15], [16]. However, this is the first time that the case of electrolysis based renewable hydrogen is investigated. This study can therefore be seen as a replication study that is conducted in a new setting.

## 2 Methodology

To answer the main question of the paper, we applied a proven three stage method. In stage 1, a list of factors that can distinguish PEM and alkaline was drafted from the literature that has reported on this technology battle [4], [17], [18], [19], [20], [21]. Furthermore, two interviews with experts were conducted. The first expert is an Innovation Technologist expert on electrochemical engineering. The second expert is a Research, Design and Innovation Specialist with an expertise in electrochemistry. If a factor was mentioned either explicitly or implicitly in the literature or in one of the interviews, it is seen as a relevant factor that would be used for the next stage.

In stage 2, the weight of each of the relevant factors was analysed using the best worst method (BWM) interviewing six experts. The BWM will be explained in the last part of this section. In addition to the previously mentioned second interviewee, five more were consulted at this stage. Finally, in stage 3, three experts were interviewed. They were asked whether they could explain the weight of factors. Interviews were conducted in the Netherlands as this is one of the countries presenting the most progress and investment in the sector [4] and, therefore, a high availability of experts. Table 1 presents an overview of the interviewees' background, expertise, function. The final column indicates the research stage at which they were interviewed.

**Table 1:** Summary of each respondents' background and timeline of intervention in the answers gathering process.

	Background	Expertise	Function	Stage
1	Academia	Electrochemical engineering	Assistant Professor electrochemical engineering	1
2	Industry	Electhrochemistry	RD&I Specialist	1, 2
3	Industry	Hydrogen systems, electrical engineering	Development process engineer	2
4	Industry	Business development, energy conversion, energy carriers, asset management	Co-owner of company that develops water electrolysis integrated in wind turbines	2
5	Industry	Hydrogen, supply chain management	Energy analyst	2
6	Industry	Business development, renewable energy	Director business development	2
7	Industry	RD&I, Chemical engineering, hydrogen business development	RD&I OPS manager	2
8	Industry	RD&I	Green hydrogen program manager	3
9	Industry	RD&I, business development	Director of hydrogen section	3
10	Academia	Hydrogen, energy	Professor Future energy systems	3

### 2.1 BWM

BWM is a multi-criteria decision making approach [22], [23] which can be used to allocate weights to different factors. This approach enables better results compared to alternative multi-criteria decision making approaches because it allows researchers to obtain pairwise comparison (comparing entities in pairs to judge which of the two is preferred) for data in a structured manner [22]. Another advantage of this method is that it requires less data as an input than similar methods. Thanks to these qualities, the BWM method is successfully used to evaluate complex situations pertaining to supplier segmentation and selection, innovation management, university-industry collaboration as well as water resource management [24], [25], [26], [27]. The BWM consists of five steps as first described by the developer of the method [22]:

• Step 1: Establish a set of criteria.
  These criteria represent different factors, related to the given standards analysis, found in the literature and through interviews $c_{1},c_{2},c_{3},...,c_{n}$, where *n* represents the last criterion of the ones considered (e.g., if 7 criteria are considered, $c_{n}$ would be $c_{7}$).
• Step 2: Select the most and least desirable criteria.
  At this stage, every respondent is required to select the best (most desirable) and worst (least desirable) criteria, which can be any out of the previously listed ones. These can be respectfully represented as $c_{b},c_{w}$.
• Step 3: Evaluate the degree to which the best criterion prevails over the others.
  The respondent is required to express his preference of the chosen criteria compared to the others on a scale from 1 (equal preference) to 9 (extreme preference). Here, the *Best-to-Others* vector is created, e.g., $A_{C}=(a_{C}1,a_{C}2,...,a_{C}n)$ where $a_{C}j$ indicates the preference of the best criterion *C* over other criterion *j*. *j* Represents a given criterion chosen for comparison with the best one (e.g., if the best criterion is compared to the fifth criterion, this would be $a_{c}5$). The 1-9 scale was found to be the optimal methodology to follow in te literature [22], [23], [24], [25], [26], [27].
• Step 4: Evaluate the degree to which the worst criteria under performs compared to the others.
  Similarly to the previous step, the respondent is required to express his preference of all criteria over the worst criterion. The same mathematical logic considering numbers from 1 to 9 is applied. Here, the *Others-to-Worst* vector is created, e.g., $A_{Z}={\left(a_{1}Z,a_{2}Z,...,a_{n}Z\right)}^{T}$ where $a_{j}Z$ indicates the preference of any criteria *j* over the worst criterion *Z*.
• Step 5: Calculate the optimal weights ($w_{1}^{⁎}$, $w_{2}^{⁎}$,..., $w_{n}^{⁎}$).
  The optimal weight for one criterion is one where, for each pair $w_{C}/w_{j}$ and $w_{j}/w_{Z}$, there is that $w_{C}/w_{j}=a_{C}j$ and $w_{j}/w_{Z}=a_{j}Z$. For this condition to be satisfied for every *j*, there needs to be a solution where the maximum absolute differences for every *j* are minimized. Hence, the following problem results (considering non-negativity and sum conditions for every weight):
  $min,max_{j}\{|\frac{w_{C}}{w_{j}}-a_{C_{j}}|,|\frac{w_{j}}{w_{Z}}-a_{J_{Z}}|\}$
  Such that
  $\sum_{j}w_{j}=1$
  $w_{j}\geq 0$, for all *j*
  This can be rewritten as:
  minξ
  such that
  $|\frac{w_{C}}{w_{j}}-a_{C_{j}}|\leq \xi$, for all *j*
  $|\frac{w_{C}}{w_{z}}-a_{j_{z}}|\leq \xi$, for all *j*
  $\sum_{j}w_{j}=1$
  $w_{j}\geq 0$, for all *j*
  Where $w_{C}/$ represent the weight of the best criteria, $w_{Z}/$ the weight of te worst criteria and $w_{j}/$ the weight of a specific criteria
  By solving the latter, the optimal weights ($w_{1}^{⁎}$, $w_{2}^{⁎}$,..., $w_{n}^{⁎}$) and the optimal value $\xi^{⁎}$ are achieved.
  By consulting the third expert (Table 2) and the literature the technologies have been given a performance score in all categories on a scale from 1 to 5. The score is then multiplied with the global weights, and finally the scores are added to give a final score for each technology.

**Table 2:** Comparison of technical characteristics of competing technologies.

	Alkaline	PEM
Advantages	Well established technology, non noble catalysts, long-term stability, relative low cost, stacks in the MW range, cost effective [19]. Technology: Oldest and Well established; Cost: Cheapest and effective; Durability: Long term; Stacks: MW range; Efficiency: 70%; Most mature electrolysis technology [21].	High current densities, high voltage efficiency, good partial load range, rapid system response, compact system design, high gas purity, dynamic operation [19]. Current density: High; Voltage efficiency: High; Load range: Good partial load range; System Design: compact; Degree of Purity: High gas purity; Dynamic: high dynamic operation; Response: rapid system response [21].

Disadvantages	Low current densities, crossover of gases (degree of purity), low partial load range, low dynamics, low operational pressures, corrosive liquid electrolyte [19]. Current Density: Low; Degree of Purity: Low (crossover of gases); Electrolyte: Liquid and Corrosive; Dynamics: Low dynamic operation; Load range: Low for partial load; Pressure: Low operational pressure [21].	High cost of components, acidic corrosive environment, possibly low durability, commercialization, stacks below MW range [19]. Technology: New and partially established; Cost: High cost of components; Catalyst type: Noble catalyst; Corrosion: acidic environment; Durability: comparatively low; Stack: Below MW range; Membrane: limited and costly; Commercialization is

## 3 Theoretical exploration

Various scholars have studied how technological innovation becomes dominant. Some scholars take the perspective of the industry in which a dominant technology is established. For example, evolutionary economists argue that technology dominance cannot be explained ex ante but only ex post. They argue that through path dependencies a technology can rise towards dominance [28], [29]. Technology evolves gradually into the dominant design [30], [31]. This is a process of experimentation where different trajectories are followed [31].

Other scholars take the viewpoint of a firm that attempts to achieve dominance with its technology and set a dominant design or dominant standard. For example, technology and innovation management scholars argue that a promoter of a technology can achieve success with its technology by strategic manoeuvring [13] such as setting a low price or pre-announcing a technology. These strategies are especially relevant in industries that are characterized by increasing returns to adoption.

Finally, scholars have approached this topic from the perspective of the firm or consumer that has to make a decision for one or the other technology to adopt [32]. These scholars utilize insights from institutional theory [33] and network economics [34], [35] to explain such choices. For example, institutional pressures can lead a consumer to choose for a particular technology because of peer pressures. Additionally, when a technology increases in value the more it is being used by others (direct network effects [34], [35]) it will gather more attraction making users more prone to adopting that technology.

Suarez [13] distinguishes five different phases in which battles for standard dominance are fought throughout the technology life cycle. In this case, the battle in question can be placed in the third stage: creating the market, as the first commercial products have already been launched on the market, and the focus has moved from technological factors to market factors. This means that technology is mature and market dominance is assumed to be dependent upon the strategies that are applied by the firms that back up the technologies.

## 4 Practical exploration

The most mature technologies are alkaline electrolysis and PEM [4]. On paper, both technologies have pros and cons, but both are still under development and are yet to be tested on a larger scale. PEM electrolysis entails higher operational costs, since the latter is more sensitive to water impurities [4]. On the other hand, alkaline electrolysers present a lower power consumption. Nonetheless, alkaline electrolysers do entail a higher plant footprint, which can be undesirable especially in the field of renewable hydrogen. This is also due to the fact that alkaline electrolysers are an older technology compared to PEM.

The most important factors differentiating both electrolyser technologies are investment and operational costs as well as the ability to operate at high current densities, variable partial load, overload, and on/off conditions [4]. Alkaline electrolysis is an older technology which performs better in terms of costs (Table 2). Indeed, the newer type of technology presents high costs of components. However, PEM electrolysis, performs significantly better when it comes to coping with intermittent power sources [4]. Another set of factors to assess the performance of both technologies is capital costs, lifetime, efficiency and environmental impacts [18].

Most experts identify Alkaline electrolysis as the most suitable technology at current production levels. However, PEM is expected to outperform Alkaline in 2030 when production is scaled up [18]. In the case of electrolysis technology, the main environmental problem is mining key components as well as the health and contamination issues related to the use of nickel and platinum [18]. Another study, values most importantly the costs, the current densities, the load range, the MW range of stacks, the purity of the gas and the rapidity of the system response, as well as the level of current densities and the voltage efficiency primarily [19]. As can be seen in Table 2, PEM electrolysis present good partial range when compared to alkaline.

Stemming from another study, the main factors to evaluate are: current density, work pressure (MPa), operating temperature (^∘^C), hydrogen purity (%), raw components, corrosion, operating characteristics, structural features, volume, weight, manufacturing cost and lifetime [20]. According to this study, on one hand, Alkaline is a more mature technology, and is suitable for the large-scale manufacturing of hydrogen, even though it has a slow startup and suffers from complicated maintenance and complicated machinery [20]. PEM on the other hand, according to the same paper, has a fast startup, no corrosion, simple maintenance and few components. The problem of PEM is high manufacturing costs [20].

Finally, according to yet another academic paper, some of the factors identified are: how new the technology is, the cost, the catalyst type, the degree of purity, the dynamics of system response, the load range, the pressure, the efficiency, the durability, the range of the stacks, the voltage efficiency and the compactness of the system designed [21].

Stemming from the literature review, it is possible to separate factors into four aggregate categories as it is done in the framework of Schmidt et al. [18]. These four categories are identified as: capital costs, lifetime, efficiency and environmental impacts. Factors included in capital costs are initial investment and operational costs. Lifetime consists of the amount of time an electrolyser can stay in operation. A factor closely related to lifetime, is durability, which is defined by the amount of maintenance needed for the electrolyser to operate. Efficiency is identified as the ability of the electrolyser to achieve the highest input possible with the lowest input possible in intermittent current conditions. Efficiency is interrelated with the ability of the electrolyser to operate in the context of intermittent current. Finally, environmental impacts, is also a factor of significance. It is related to many other variables, the most noteworthy one being the materials used to produce the electrolysers. The delineated factors deemed to be of crucial importance stemming from literature review in this technology battle have been grouped and listed together in Table 3. The next steps will consist in performing interviews to experts in the field, reassess the importance of different factors and thereafter apply the BWM methodology to gain insightful results.

**Table 3:** List of factors used for the BWM interviews.

Factor	Explanation
Price	The costs involved in purchasing and implementing the technology. The latter relates to, e.g., operational or manufacturing costs [17], [18]. The lower these costs, the higher the chance that the novel technology will be adopted en masse and reach dominance.

Stack size	Compactness of the electrolyser stacks [20], [21]. Compact electrolyser stacks can have an advantage over non-compact electrolysers, as some projects are in confined spaces.

Flexibility	Ability to operate with variable partial load, overload, load range, on/off conditions and rapidity of the system response [17], [19], [21]. In most future scenarios for hydrogen, electrolysers must be able to operate flowing the wind profiles. Flexibility is key and could determine which technology will reach dominance

Energy consumption	(Voltage) Efficiency and current density [18], [19], [21]. The more efficient and the denser, the higher the chance that the technology will be adopted and reach dominance.

Lifetime	The number of years electrolysers generally function and the durability [18], [20]. This will have a positive effect on technology dominance.

Safety	The risk that critical failures occur during operation. When that risk is high and safety is therefore low, the chances will increase that the technology will not be adopted and will not become dominant. The risk that critical failures occur during operation.

Materials used	The extent to which the raw materials that are used to build electrolysers are available and do not have a negative effect on the environment which positively affects technology dominance [18], [20].

## 5 Results

Table 3 shows the relevant factors that affect technology dominance for renewable hydrogen-based electrolysis. Most factors were identified in the literature and one factor, safety, was mentioned by an expert.

The results of the BWM are displayed in Table 4. The factors can now be ranked from best to worst as follows: Price, Safety, Energy consumption, Flexibility, Lifetime, and Stack size and Materials used tie for least important factors. Table 5 shows the performance of PEM and alkaline technologies. The total scores indicate that alkaline (3,253) has a slight advantage over PEM (2,888).

**Table 4:** BWM Results.

Expert	Price	Stack size	Flexibility	Energy consumption	Lifetime	Safety	Materials used	KSI
1	0.270	0.163	0.163	0.109	0.163	0.024	0.109	0.057
2	0.361	0.073	0.109	0.145	0.062	0.218	0.032	0.076
3	0.332	0.104	0.028	0.208	0.138	0.052	0.138	0.083
4	0.208	0.052	0.332	0.138	0.138	0.028	0.104	0.083
5	0.135	0.060	0.135	0.203	0.081	0.335	0.081	0.083
6	0.140	0.060	0.211	0.105	0.105	0.348	0.031	0.073
7	0.351	0.085	0.030	0.213	0.107	0.142	0.071	0.076
Average	0.257	0.081	0.144	0.160	0.114	0.164	0.081	0.074

**Table 5:** Ranking of the different factors.

	PEM		Alkaline	
Factor	Performance	Score	Performance	Score

**Price**	2	0.513	4	1.026
**Stack Size**	5	0.405	2	0.0.162
**Flexibility**	4	0.576	3	0.432
**Energy Consumption**	3	0.481	4	0.641
**Lifetime**	3	0.341	3	0.341
**Safety**	3	0.491	2	0.328
**Materials used**	1	0.081	4	0.323
**Total**		2.888		3.253

## 6 Discussion

### 6.1 Interpretation of the results

This section will interpret the results making use of the literature, the three additional interviews that were conducted in stage 3 of the research and common sense. The most important factor was price, followed by safety and energy consumption. This is consistent with the findings from Carmo et al. [17] that high investment and operational costs currently act as a barrier for meeting the future demand of water electrolysers, which means these costs must be reduced. This highlights the importance of affordable electrolyser prices and operating costs to enable their large-scale implementation on a global level.

Safety was ranked second in terms of importance. Indeed, a high safety level is essential to facilitate the large-scale implementation of a technology. According to some experts, the safety levels of PEM and alkaline electrolysers are very similar, which may explain why the factor is not ranked higher. Some of the experts were even surprised that it was seen as the second most important factor because some claimed that the difference in safety levels between the two technologies is too small to impact battle for dominance. Others claimed it should have a higher rank as it still needs research. According to experts interviewed: “Safety is a factor that needs more research in general. From a manufacturer point of view, it is the most important factor.” Safety standardisation may follow a de jure standardisation, which is led by the cooperation between different stakeholders, or a de facto standardisation, which is led by the competition between different companies [36]. In the case of the competing safety standards in electrolyser technologies for renewable hydrogen generation, cooperation may be led by governments and regulatory bodies, while competition can be found among eclectrolyser manufacturers.

The third most important factor, energy consumption, highly impacts the price of manufacturing hydrogen as a lower energy consumption would entail a lower cost of production for the hydrogen produced. During the third round of interviews, the experts confirmed that the factors price and energy consumption are crucial in the battle for dominance. According to an expert, “the most crucial factor is the levelized cost of hydrogen” and many of the factors identified in the research are interdependent and make up the levelized cost hydrogen. However, the order of importance of the chosen factors and their impact on the levelized cost of hydrogen is highly dependent on the location of the electrolysers. For this research mostly experts working in northern Europe were interviewed. In Northern Europe electricity is expensive and renewables are scarce. Therefore, the energy required to produce hydrogen is a crucial component in the price. In countries with abundant clean electricity, in the south of Europe or northern Africa, energy consumption is less important, as it can be several times cheaper than in the Netherlands. If the same research was conducted outside of Europe, energy consumption would have received a lower rank, according to one of the experts.

Flexibility was ranked as the fourth most important factor, since it affects the ability of electrolysers of operating with intermittent energy sources. However, according to some interviewees, although in theory PEM electrolysers have a higher flexibility, in practice, pressurized alkaline electrolysers can have a flexibility comparable to that of PEM electrolysers.

### 6.2 Theoretical contributions and practical implications

This paper contributes to the literature on dominant designs and standards battles [13] in several ways. First, we investigate factors for technology dominance for a case that has never been studied before; technology dominance for electrolysis based renewable hydrogen production. This research can therefore be seen as a replication study in a new context. Second, we arrive at various new factors for technology dominance that have not been mentioned before including energy consumption and safety. Third, the paper shows that price is an important factor for technology dominance and thereby confirms earlier findings. Finally, the paper shows that price is important in the third stage of the technology life cycle which confirms the earlier findings from Suarez [13] who argues that strategic manoeuvring (of which setting a price is an element) is important in the third stage. We provide empirical evidence of that statement.

This research may be useful for managers and policy makers who want to understand which factors can be influenced in order for a given electrolyser technology to reach dominance. Furthermore, policy makers could in theory affect the preference of managers for one technology or another by increasing safety requirements. If efforts are made to improve in the most important factors, these developments could completely change the road to technology dominance. This could be achieved through innovation, but also by strategically lowering the price, as it is the most important factor. In Table 6 the results of PEM achieving equal price levels, ceteris paribus, compared to alkaline are shown. Improving the Price performance factor by 2 already would give PEM technology the upper hand in this technology battle. In comparison, improving the factor materials used, which is one of the lower ranking factors, from 1 to 5 would not result in dominance for PEM. These examples show the importance of strategically investing resources for improving technology in key factors can make crucial impact.

**Table 6:** Equal price performance score, ceteris paribus.

	PEM		Alkaline	
Factor	Performance	Score	Performance	Score

**Price**	*4*	*1.026*	4	1.026
**Stack Size**	5	0.405	2	0.0.162
**Flexibility**	4	0.576	3	0.432
**Energy Consumption**	3	0.481	4	0.641
**Lifetime**	3	0.341	3	0.341
**Safety**	3	0.491	2	0.328
**Materials used**	1	0.081	4	0.323
**Total**		*3.401*		3.253

### 6.3 Limitations and recommendations for further research

Flexibility, as defined by industrial actors, can be equated to the ability to operate in the context of intermittent current. According to some of the experts, this factor has a limited influence since pressurized alkaline electrolysers could reach the same flexibility of PEM electrolysers. Meanwhile, other experts see it as a crucial factor since electricity production is also becoming increasingly flexible. The need to align hydrogen production with electricity consumption is now embedded in EU regulation. The revised Renewable Energy Directive (RED II) explicitly states that hydrogen can only be classified as renewable if producers can prove temporal correlation between hydrogen production and RES production. Therefore, if an electrolyser has low flexibility, it might not be able to produce renewable hydrogen [37].

Energy consumption is the industrial actor's equivalent of efficiency. Durability was identified as an important factor by researchers. It is highly intertwined with lifetime, which was identified as an important factor by experts. Additionally, environmental impact was not considered by experts. They argue that the materials used is the biggest factor when it comes to the environmental impact of electrolysers. This is also consistent with what was found in Schmidt et al. [18].

According to industry experts, it is difficult to compare the performance of PEM and alkaline electrolysers since it is highly context dependent. For instance, in plants operating continuously, energy consumption is more important than the price. This, however, is not true for electrolysers with a low utilisation.

Future research may also study the extent to which the factors for technology dominance for renewable hydrogen-based electrolysis are interrelated. One expert noted: “Most factors [present in the BWM matrix] are interdependent […] only flexibility is an independent variable”. Additionally, future research could study the extent to which the importance of one factor over another might be dependent on other contextual factors. For example, factor importance might be dependent on the geographical location of the plant. Indeed, in plants where renewable energy sources are abundant, the price factor increases in importance compared to the efficiency factor. On expert noted: “In Africa, price would be more important than efficiency since solar power can be cheap and abundant there”. However, in places where renewable energy sources are scarcer, the prioritization of price over efficiency is less significant. In this case there are multiple technological fields in which PEM and alkaline battle for dominance. In accordance to the characteristics of technological fields as explained by Suarez [13], in different technological fields, there are different technological trajectories that compete for dominance that depend in part on the structure and dynamics of specific technological fields.

Pertaining to the use of rare materials in PEM electrolysers, the use of nano depositions could significantly reduce the volumes of materials needed to produce an electrolyser. One expert even added that “there will most likely be no restrictions to scale up PEM technology”. This paper has focused on the two most dominant technologies for renewable hydrogen-based electrolysis. However, there is a promising third technology emerging: Anion Exchange Membrane (AEM) and solid oxyde electrolysers. AEM can basically be seen as the successor of alkaline technology. Although it is still in an early stage of development it is seen by some as a potential gamechanger while others argue that it is too late for the technology to catch up with PEM and alkaline, as they are rapidly scaling up and becoming cheaper. Future research could study the extent to which new technologies can battle against incumbents. According to the experts that we have interviewed, it is important to note that these technologies are at a great disadvantage in this technology battle. Indeed, compared to PEM and alkaline electrolysers, they face grave challenges in scalability. As a final note, the experts are among the top experts in this area, so their opinion really matters. The main purpose of the paper is to get some initial insights on the case and show how a method like BWM can help us in this regard. For this purpose a survey of 10 experts is sufficient. Future research should collect data from other (and more) experts to get more insights, and make the results more reliable.

## 7 Conclusion

The most important factors appear to be: Price, Safety, Energy consumption, Flexibility, Lifetime, Stack size and Materials used. Factors that directly influence the total cost of hydrogen, price of the electrolyser and energy consumption, are identified as being crucial in the battle for dominant design for electrolysis. Indeed, when considering these factors, alkaline outperforms PEM. Even though this gives alkaline electrolysers a slight advantage in the battle for dominant design, it is too early to make predictions. The hydrogen industry and the technology are still being developed. As the industry matures and scalable technologies develop, it is likely that the performance of alkaline and PEM electrolysers, vis-à-vis the factors identified, might change. To become the dominant design, producers and researchers of the technologies should focus on improving the highly ranked factors. Alkaline now has an advantage, however PEM has more room for improvement.

## CRediT authorship contribution statement

Bernhard van Haersma Buma: conceived and designed the experiments; performed the experiments; analyzed and interpreted the data; wrote the paper

Marco Peretto, Ziad Matar: conceived and designed the experiments; analyzed and interpreted the data; wrote the paper

Geerten van de Kaa: analyzed and interpreted the data; contributed reagents, materials, analysis tools or data; wrote the paper

## Declaration of Competing Interest

The authors declare that they have no known competing financial interests or personal relationships that could have appeared to influence the work reported in this paper.

## Data Availability

Data will be made available on request.
